# Cybersecurity challenges in energy sector (virtual power plants) - can edge computing principles be applied to enhance security?

**DOI:** 10.1186/s42162-021-00139-7

**Published:** 2021-03-31

**Authors:** Sampath Kumar Venkatachary, Annamalai Alagappan, Leo John Baptist Andrews

**Affiliations:** 1 Grant Thornton, Plot 50370, Acumen Park, Fairgrounds, Gaborone, Botswana; 2 Department of Network and Infrastructure Management, Faculty of Engineering and Technology, Botho University, Gaborone, Botswana; 3 Department of Information Technology, Faculty of Engineering and Technology, Botho University, Gaborone, Botswana

**Keywords:** Edge computing, Virtual power plants (VPP), Distributed energy resource (DER), Security architecture, IDS, Authentication and authorisation, Privacy

## Abstract

Distributed generators (D.G.’s) enable us to generate, supply and be self-reliant on power while also allows us to supply power to meet the demand through virtual power plants. The virtual power plants also help us analyse, control, optimise, and help bridge the gap of demand and supply in these vast energy requirements. With this also comes challenges associated with securing physical systems, data protection and information privacy. Recent technological advancements have aided cybercriminals to disrupt operations by carrying out deliberate attacks on the energy sector. Though security researchers have tried to mitigate the risks, vulnerabilities, and it remains a challenge. This paper aims to present a comprehensive Edge-based security architecture to help reduce the risks and help secure the physical systems and ensure privacy and data protection.

## Introduction

**Fig. 1:**
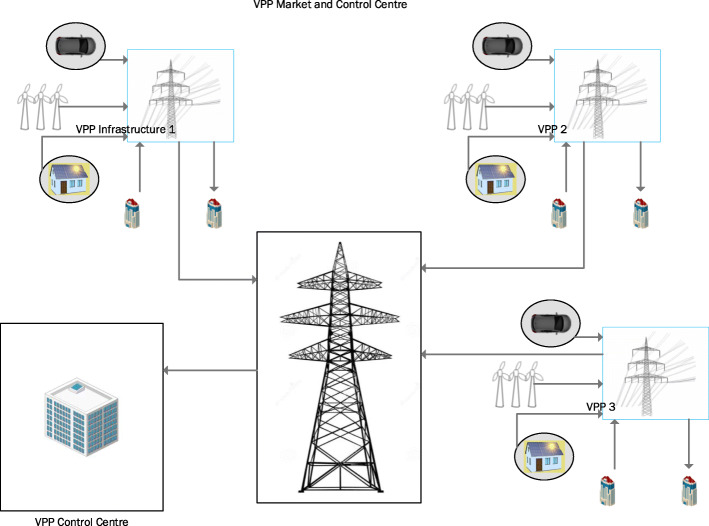
VPP Energy System

Virtual Power Plants (VPP), Smart Grids (S.G.). Virtual Power Plant, “As its name infers, a virtual power plant does not exist in the solid and-turbine sense. It utilises the smart grid infrastructure to integrate little, divergent energy assets as though they were a single generator. Pretty much any energy source can be connected up. (Kumagai, 2012). Moreover, the energy can likewise add to a virtual power, not plant’s capacity” The point of VPP’s is to distributed appropriated energy assets over the virtual energy pool. (Fig**.** 1) shows a brief overview of a Virtual Power Plant. Unlike traditional energy systems, the energy generation is not centralised in a remote location and then transmitted in a complex network but instead generated in small individual distributed areas. In this, a consumer can become a prosumer and supply the excess energy generated back to the grid. The traditional model, though, is cost-effective the outreach of the model in third world countries pose a problem where the majority of the population have no access to energy. This problem technically can be addressed by using distributed energy networks and effectively exercise control through a VPP operator. It is expected that by 2035–2040 the electricity system will mostly constitute decentralised IoT devices effectively communicating through virtual power plants and distributed energy systems. In short, electricity will be digital.

This growing deployment of small prosumers also poses a problem in the grid systems which also needs to adopt a decentralised approach to reduce the complexity and overcome the increasingly new challenges in management (Pop et al., 2019). These deployments pose a different set of problems in the form of efficiency in integration, energy supply security, continuity. Assuming the energy generated is not consumed by the consumer in the resource, it could also technically lead to over-voltage problems, losses, transformer ageing and efficiency.

The future energy networks will relate to advance distribution and management systems, including using data relating to grid monitoring, control, sensors, load balancing requirements, environmental parameters etc. (Rennie, 2019). The range of data shared between transmission and distribution, system, grid operators, consumers, prosumers, aggregators are enormous. Most of these systems will also be using intelligent control systems, distributed intelligence employing A.I. This will also help enhance consumers with improving capabilities, reporting and managing infrastructure.

### Edge virtual power plants

The term edge computing is relatively a new concept, though very similar to other computing terminologies in use. Edge computing refers to simple process operations carried out close to the origins of data. In simple terms, the processes can be done on the devices rather than on the servers, increasing the processing speed. Therefore, it is possible to offload a few resource-hungry tasks to the new edge layer, thereby reducing the impact on resource-constrained resources. The application of edge technology in virtual power plant technically involves optimising resources through machine learning algorithms. As more and more DER systems integrate, the data must be processed balloons, requiring more processing power. Since each of these devices communicates with the IoT devices in the household, the information processed can be done locally (Rennie, 2019).

Traditional VPP’s mostly are controlled centrally, and the information is collated and transmitted to these central units through a communication environment including 5G technologies (Jaber et al., 2016; Khodashenas et al., 2016) (Zaho & Gerla, 2019). 5G communication technologies are said to noted to have privacy issues in a centralised environment (Cai et al., 2019; Cai & Zheng, 2019; Tian et al., 2019), leading researchers to suggest distributed control methods (Chen et al., 2018a, b, c; Cai & He, 2019; Huang et al., 2019). The advancement of technology has also led to research on edge computing for processing information and control. (Chen et al., 2018a, b, c; Chen et al., 2018a, b, c). The rise of A.I. and cognitive computing (Chen et al., 2019) has paved the way for applying mathematical tools to improve processes and efficiency, which are popularly termed as Edge Intelligence (Zhou et al., 2019; Rausch & Dustdar, 2019). Due to this huge demand for processing on the edge nodes, edge computing applies the A.I. to enhance the processing speeds. The application of edge intelligence computing requires a huge communication network and bandwidth. As VPP is also a combination of distributed networks, some of these problems apply. Some of these problems have been effectively addressed to minimise the costs and reduce the communication environment by Li et al. (Li et al., 2018).

These dependencies on the ICT infrastructure also has potential cybersecurity threats. Since the operations are widespread and network-based with individual endpoints, the attack surface in a virtual power plant is vast since the core of the processes is from the control centre. The threat actors multiply manifold due to the different RTUs and SCADA gadgets. Any vulnerability in a single system is a gateway for hackers to get into the network. It can be noticed from the data analysed that the critical infrastructure services are frequently being targeted with malware or ransomware with a motive for financial gain or disruption. (Venkatachary et al., 2017; Venkatachary et al., 2018a; Venkatachary et al., 2018b). They are thus providing a way for enhancing security mechanisms across the network. Therefore, this new edge concept also offers the opportunity to deploy new based security solutions on the end devices, thus optimising performance. (Montero et al., 2016; Mach et al., 2017; Errabelly et al., 2017; Tao et al., 2017; Hsu et al., 2018).

Against this backdrop, this paper aims to provide an insight into various cybersecurity threats that emanate from these advance technological applications. Section 2 provides a detailed insight into cybersecurity trends and facilities attacked, and the need for better security. Section 3 discusses at length the proposed Edge-based solutions towards enhancing security in virtual power plants. Section 4 and 5 provides a detailed discussion and conclusions.

## Cybersecurity trends and the edge centric architecture for VPP

**Fig. 2:**
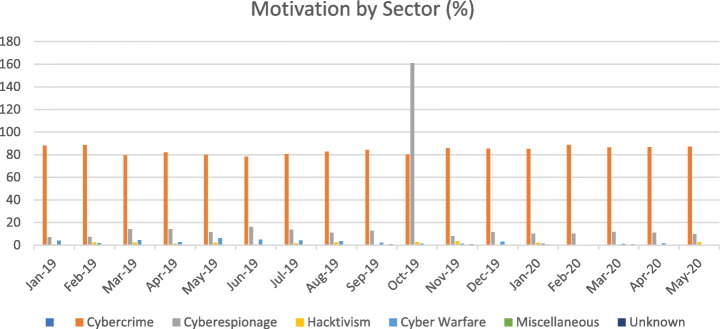
Motivation trends for Attacks from 2019 to 2020

**Fig. 3:**
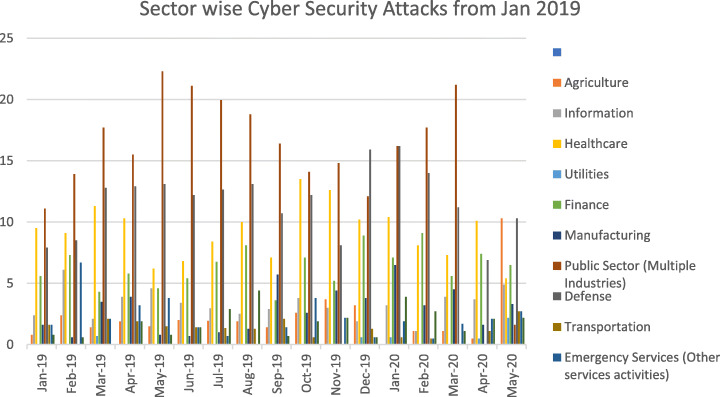
Sectors targeted from 2019 to 20

Among the sectors, the energy sector is one of the most targeted sectors in recent times. The motivation of the attackers has changed over time. Though the primary motivation still remains money, other motivations like cyber warfare and causing disruptions have also witnessed an increase Figs. 2 & 3, and Table 1 outlines the basis and the sectors targeted. As can be seen, the trends during 2020 have changed an increase in health care facilities being targeted more than other industries. Given the vulnerabilities in the firmware of different types of equipment and addressing the vulnerabilities through patch mechanisms a nightmare for security firms, the energy sector is a primary motivator for cyber-attacks. According to data by Kaspersky labs, the attack vectors included DDos, Java Script, BAT, V.B. Script, Python, Word on the platforms (Kaspersky Labs, 2020).

**Table 1:** Cybersecurity incidents on Critical Infrastructure Services from 2019 to May 20

Motivation by Sector	Jan-19	Feb-19	Mar-19	Apr-19	May-19	Jun-19	Jul-19	Aug-19	Sep-19	Oct-19	Nov-19	Dec-19	Jan-20	Feb-20	Mar-20	Apr-20	May-20
Cybercrime	88.1	88.5	79.4	81.9	80	78.2	80	82.6	84.3	80.1	85.9	85.4	85.2	88.7	86.6	86.8	87
Cyberespionage	7.1	7.3	14.2	14.2	11.5	16.3	13	11.2	12.9	161	8.1	11.5	10.2	10.2	11.7	11.1	9.8
Hacktivism	0.8	2.4	2.1	1.3	2.3	0.7	1.6	2.5	0	2.6	3.7	0	1.89	0.5	0	0.5	2.7
Cyber Warfare	4	1.8	4.3	2.6	6.2	4.8	4.5	3.7	2.1	1.3	1.5	3.2	1.32	0.5	1.1	1.6	0.5
Miscellaneous	0	0	0	0	0	0	0	0	0	0	0	0	0.57	0	0	0	0
Unknown	0	0	0	0	0	0	0	0	0.7	0	0.7	0	0.19	0	0.6	0	0
Critical Infrastructure Services	Jan-19	Feb-19	Mar-19	Apr-19	May-19	Jun-19	Jul-19	Aug-19	Sep-19	Oct-19	Nov-19	Dec-19	Jan-20	Feb-20	Mar-20	Apr-20	May-20
Agriculture	0.8	2.4	1.4	1.9	1.5	2	1.9	1.9	1.4	2.6	3.7	3.2		1.1	1.1	0.5	10.3
Information	2.4	6.1	2.1	3.9	4.6	3.4	2.9	2.5	2.9	3.8	3	1.9	3.2	1.1	3.9	3.7	4.9
Healthcare	9.5	9.1	11.3	10.3	6.2	6.8	8.4	10	7.1	13.5	12.6	10.2	10.4	8.1	7.3	10.1	5.4
Utilities			0.7									0.6	0.6			0.5	2.2
Finance	5.6	7.3	4.3	5.8	4.6	5.4	6.7	8.1	3.6	7.1	5.2	8.9	7.1	9.1	5.6	7.4	6.5
Manufacturing	1.6	0.6	3.5	3.9	0.8	0.7	1	1.3	5.7	2.6	4.4	3.8	6.5	3.2	4.5	1.6	3.3
Public Sector (Multiple Industries)	11.1	13.9	17.7	15.5	22.3	21.1	19	18.8	16.4	14.1	14.8	12.1	16.2	17.7	21.2		1.6
Defence	7.9	8.5	12.8	12.9	13.1	12.2	12	13.1	10.7	12.2	8.1	15.9	16.2	14	11.2	6.9	10.3
Transportation	1.6		2.1	1.9	1.5	1.4	1.3	1.3	2.1	0.6		1.3	0.6	0.5		1.1	2.7
Emergency Services (Other services activities)	1.6	6.7	2.1	3.2	3.8	1.4	0.7		1.4	3.8	2.2	0.6	1.9	0.5	1.7	2.1	2.7
Energy	0.8	0.6		1.9	0.8	1.4	2.9	4.4	0.7	1.9	2.2	0.6	3.9	2.7	1.1	2.1	2.2

The traditional approaches to handling cybersecurity using firewalls and cryptography incidents are outmoded due to the variety and complexity of attacks in recent times. The complexity of cybersecurity attacks in the form of disabling, tampering, reprogramming the control systems can lead to malfunctions, unavailability of system services during critical operations, which could lead to other consequences in the form of human life. (Venkatachary et al., 2018a; Venkatachary, 2018b; Venkatachary et al., 2020) In short, the cybersecurity attacks in the recent past has undergone a sea change. Some notable examples are black energy, Stuxnet and so on (Symantec, 2009; Symantec, 2011; Liu et al., 2012).

### Overview of cyber attacks and the need for better security to secure energy systems

With the rise in energy demand, the distributed generators play a vital role in bridging the gap between demand and supply, securing the devices gain prominence. Security in device controllers is often overlooked as it is mostly isolated and tied to the infrastructure. This poses a problem of often not getting the control devices patched, thereby exposing them to vulnerabilities and attacks. An underlying problem in securing devices is the responsibility attached to the person. Often, it is found that most operators operating these machines simply do not have the experience or expertise and the knowledge of how these I.T. systems function and vice versa applies to the I.T. personnel developing necessary patches etc. (Brook, 2018).

The complexity of the distributed generators also poses a considerable risk, unlike computers and other devices, which can be managed through upgrades and patches (Bekara, 2014). The different layers that encompass the virtual power plant are complex, and the interlinks in each layer interwinds with the other layers. The nature of architecture in VPP has many ICS devices interconnected, and the attacks can take place on any of the devices like AMI, SCADA, control and monitoring devices. Taking this into account, the entire network can be made unavailable with a single point of failure.

The number of critical infrastructures targeted across the countries is tabled in Table 2. Some notable special attacks between Jan-20 to June 2020 on the critical infrastructures is tabled in Table 3. As can be seen from the table, there is a rising volume and sophistication of the attacks on the infrastructure services and the need to safeguard the equipment, data becomes critical (Lathrop et al., 2016; Kimani et al., 2019).

**Table 2:** Targeted Cybersecurity Attacks against Critical Services, Energy Sector etc.

Year	Target Facility	Country	Agent	Impact	Ref
1982	Pipeline explosion	Russia	Malware (SCADA)	Explosion and fire.	(Zakhmatov et al., 2011)
1992	Ignalina Nuclear Power Station	Lithuania	Virus (Control System)		(Panda, 2015)
1992	Chevron (Warning System)	USA	Virus	Hacking by a disgruntled employee who left thousands of employees exposed to toxicity	(Miller & Rowe, 2012)
1994	Salt River Project	USA	Malware (Control System)	Hacking by an employee, resulting in deleting of critical files resulting in disconnecting water supply to customers	(Panda, 2015)
1997	Worcester Airport	USA	Trojan (Control System)	Air traffic Control tower system down for six hours	(Panda, 2015)
1999	Gazprom	Russia	Trojan (SCADA)	No serious consequences	(Panda, 2015)
2000	Maroochy Water System	USA	Trojan	Water spillage	(Panda, 2015)
2001	Gas Processing Plant	USA	Unknown	Service outage in the vicinity	(Panda, 2015)
2002	PDVSA	Venezuela	Worm	Production outage	(Panda, 2015)
2003	Banking Facility; Ohio Nuclear Facility		Slammer aka Sapphire	Unknown	(McGuinn, 2004; Moore et al., 2003; Poulsen, 2003)
	Railways		SoBig	23,000 miles of one railway line	(McGuinn, 2004)
2004	National Science Foundation’s Amundsen-Scott South Pole Station		Unknown	Controlling life support systems of Antarctic research station – Cyber Terror Attack	(Poulsen, 2004)
2006	L.A. Traffic Lights	USA	Malware	Reprogram the lights	(Panda, 2015)
2008	Lodz Tram attack	Poland		Control of the tram network	(Panda, 2015)
2008	Hatch Power Plant	USA	Malware	Unintentional shut down due to an update	(Desarnaud, 2017)
2009	Civil Aviation		Unknown	Data compromise; shutdown of systems	(Gorman, 2009; Mills, 2009)
2009, 2010	Natanz - Iran’s Nuclear Plant (Centrifuges)	Iran and Many countries	StuxNet	Iran’s Nuclear centrifuges were targeted. The equipment was replaced at an alarming rate.	(Naraine, 2010; Falliere et al., 2011**;** Nakashima & Warrick, 2012**;** Sanger, 2012; Langner, 2013; Kushner, 2013; Thomson, 2013)
2011	No Specific Target; Iran Nuclear Plants	Iran and Many countries	DuQu	Targeted;	(Boldizsár et al., 2011; Boldizs’ar et al., 2012; (Guilherme & Peter, 2011; Kaspersky Corp, 2011; Kaspersky Corp, 2015)
2011	Areva	France	Malware	Non-critical data theft	(Desarnaud, 2017)
2012, 2015; 2016–17; 2018–19	Saudi Aramco (UAE); RasGas (Qatar); Italy	UAE, Italy	Shamoom (alias) Disttrack; W32.Disttrack A; W32.Disttrack B;	30–35,000 Machines; D-Dos attack; FileWiper or File Eraser	(Symantec Crop, 2017; Leyden, J, 2012; NewYork Times, 2012; Perlroth, 2012; Glymin, 2017; ENISA, 2019 Symantec Corp, 2018, Trend Micro, 2018)
2012, 2015	Iran’s Nuclear Plant, Lebanon, Sriya, Sudan, etc		Flame aka Flamer, (StuxNet. Resource 207)	Approx. 1000 Machines,	(Boldizsar et al., 2012; sKyWiper Analysis Team, 2012; Alexander, 2012; McElroy & Williams, 2012; Goodin, 2012; Nakashima et al., 2017),
2013	North American Energy Companies		Dragonfly	More than 1000 energy companies in North America and Europe	(BBC, 2014; Langill, 2014; Symantec Corp, 2014)
2014	SCADA/ICS		Havex	Noticed in 146 Command and Control Server	(David, 2014; Nelson, 2016)
2014	Korea Hydro	South Korea	Malware	Reactor Manual theft; electricity and radiation exposure data	(Desarnaud, 2017)
2015	Ukrainian Kyivoblenergo		Black Energy 3	225,000 Customers left without power for 6 h on a cold December	(Lee, 2016)
	Polish Airlines		Unknown	1400 passengers grounded	(Rene, 2015)
2016	Gundremmingen (German Nuclear Power Plant)		W32.RAMNIT; Conficker	Isolated Incident on the Power Plant as the plant was isolated. The previous version of Conficker A, B, C, D, E is reported to have caused damage to 1.7 million people.	(Symantec Corp, 2011)
2020	Public Health Services	U.S.;	Ransomware	200,000 email addresses compromised, leading to many health services being impacted with ransomware. Some restored to paying the ransom.	(Kochman, 2020)
2020			AZORult; Trojan	Spreads as payload and often is used by other payloads like Djvu; primarily collects user data	(Doffman, 2020)
2020	Citrix Application Delivery Controller	Australia, Canada, Denmark, India, Sweden, Singapore U.K., USA, Switzerland, UAE-	FTP protocol exploiting vulnerability CVE-2019-1971; Algorithm Command’ file/bin/Pwd	World Wide Citrix Gateway devices were impacted affecting banking, defence, healthcare, energy, technology, higher education, legal, media	(Glyer et al., 2020)
2020	Cisco Router Exploitation Kit – Cisco RV320		Remote code execution; Metasploit Module is exploiting vul. CVE-2019-1653 CVE-2019-1652

**Table 3:** Critical Services impacted between Jan-Jun 2020

Month/Year	Target Facility	Country	Vector Type	Impact
Jan 2020	Picanol	China, Romania, Belgium	Ransomware	No information
Jan 2020	Bapco Oil	Bahrain	Wiper Attack	No severe impact.
April 2020	Water treatment facilities	Isreal	Malware	SCADA devices
April 2020	Government and Industrial Organisations	Azerbaijan’s	COVID-19; RAT, PoetRAT, Phishing	Many devices and word documents
April 2020	Energias de Portugal	Portugal	Ragnar Locker Malware (Ransomware)	1 T.B. of sensitive data with a demand for 10.9 million USD
April 2020	DESMI	Denmark	Ransomware	Impacted a few communication systems.
May 2020	Stadler	Switzerland	Ransomware	Data Theft
May 2020	Elexon	UK	Ransomware	Internal Network – Electricity Outage
May 2020	Bluescope	Australia	Ransomware	Manufacturing Operations
Jun 2020	Honda	Japan / Europe	Malware	

Security breaches are a significant concern in virtual power plant systems and could lead to colossal property losses (Sha et al., 2016) in millions. Although the overall security apparatus in the virtual power plant is challenged due to many factors involved in the design; among them, the serious is the availability. Many security features are employed to protect and ensure availability, including some of the advanced access control mechanism (Alramadhan et al., 2017), signature-based authentication (Chen et al., 2018a, b, c), homomorphic encryption (Wang et al., 2013).

## Edge centric VPP architecture

Security research on IoT-based platforms that intends to provide security solutions have been carried out by many researchers, and these efforts include Edge-based security solutions. (Mach et al., 2017; Errabelly et al., 2017; Montero et al., 2016; Hsu et al., 2018), firewall protection (Hu et al., 2014), IDS (Roman et al., 2018; Haddadi et al., 2018), IPS, privacy preservation (Lu et al., 2017; Du, 2018; Singh et al., 2017), authentication protocols (Ali et al., 2018) etc. Edge-based protection in IoT centric devices mainly is concentrated on the user (Montero & Serral-Gracia, 2016; Montero, 2015), device (Errabelly et al., 2017; Hsu et al., 2018) and endpoint security (Mukherjee et al., 2017).

The edge centric VPP architecture contains four major components, the cloud architecture, the edge layer, VPP operators, VPP end consumers/prosumers. Though resource-intensive, the cloud architecture is located far away from the virtual power plants consumers/ prosumers. Therefore the architecture cannot function efficiently, just as in IoT (Chen et al., 2016) due to its real-time application of distributing power on the grids. With the edge layer coming into effect, the components and the dynamics of the fundamental architecture changes with the Edge being the core as it can coordinate with different VPP’s while at the same time complement and ensure optimised performance of the plant. The edge layer handles the VPP consumers queries or demand response in the edge environment, thus acting as a bridge between the users and the cloud (Sha et al., 2020). Researchers have made efforts to study and design appropriate security solutions for Edge. However, as the Edge is still in its infancy stage, security is still a long way to go (Sha et al., 2016). There needs to be continuous research for enhancing general cybersecurity (Venkatachary et al., 2018a).

Edge provides a new opportunity to explore new security mechanism for a virtual power plant. Most edge designs target offloading endpoint protection on the devices to edge. This could, in turn, pose new challenges in the form of resource constraints at the Virtual Power Plant layer.

### User-centric edge-based VPP security

The key to cybersecurity is the weakest link, and the security is as good as the weakest link in the virtual power plant. With numerous VPP devices connected in a network, the prosumers/consumers access to generation, transmission & distribution of energy and data using terminal devices is imminent. When considering the security aspects, significant concerns arise. For example, the consumer may either login in from a terminal device, which is trusted and secure or from an untrusted device. In the event of the prosumer logging from an untrusted device, the security could be compensated with additional security control measures as in the case of untrusted networks. The second aspect is that the consumer may not be aware of the security or have enough knowledge to manage the infrastructure, thereby resulting in potential risk effectively. Incorporating the Edge layer in managing such as scenario is an option; however, the drawbacks could be network challenges. The additional aspect could be on the personal security of the data on the network edge (Montero et al., 2016) and the virtual guard in Edge (Montero, 2015).

**Fig. 4:**
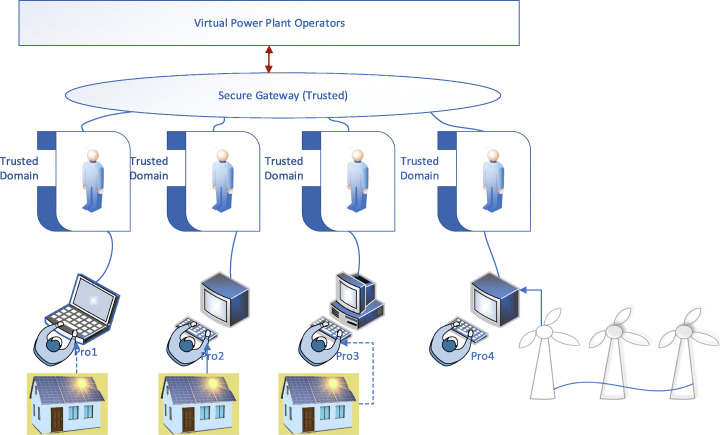
User-centric Edge-based VPP Security

Figure 4 provides a brief overview of user-centric VPP security architecture. The design incorporates a trusted domain on the edge layer. The consumers/prosumers who generate, distribute and access data incorporate additional endpoint security. This translates to user security policy such as antivirus, firewalls (Basile et al., 2010), SCADA device isolations and other inspection tools. The edge layer, which is the trusted domain, will manage the secure access to the virtual power plant operator or the virtual power transmission system operator. The trusted domain, in this case, acts as an encapsulation layer to user-specific policy. The user is verified using RVA techniques to ensure trust between the prosumer. This design is based on the Network Functions Virtualisation technology to construct the edge layer. In this way, security can effectively be managed by deploying Edge.

### Device-centric edge security for VPP

Unlike the user-centric security layer, the Device-Centric security layer is tailored to suit the prosumer or the consumer’s requirement based on the resource availability, the data sensitivity and its impact on tasks and in consideration with the security needs of the endpoint VPP devices. Erabally et al. (Errabelly et al., 2017), in their paper, discuss the device-centric edge layer security comprising of six modules that function in a synchronised manner to handle specific security challenges in the IoT systems. The individual modules in each case include a systematic analysis of security profile, protocols, simulation, communication and request handling.

**Fig. 5:**
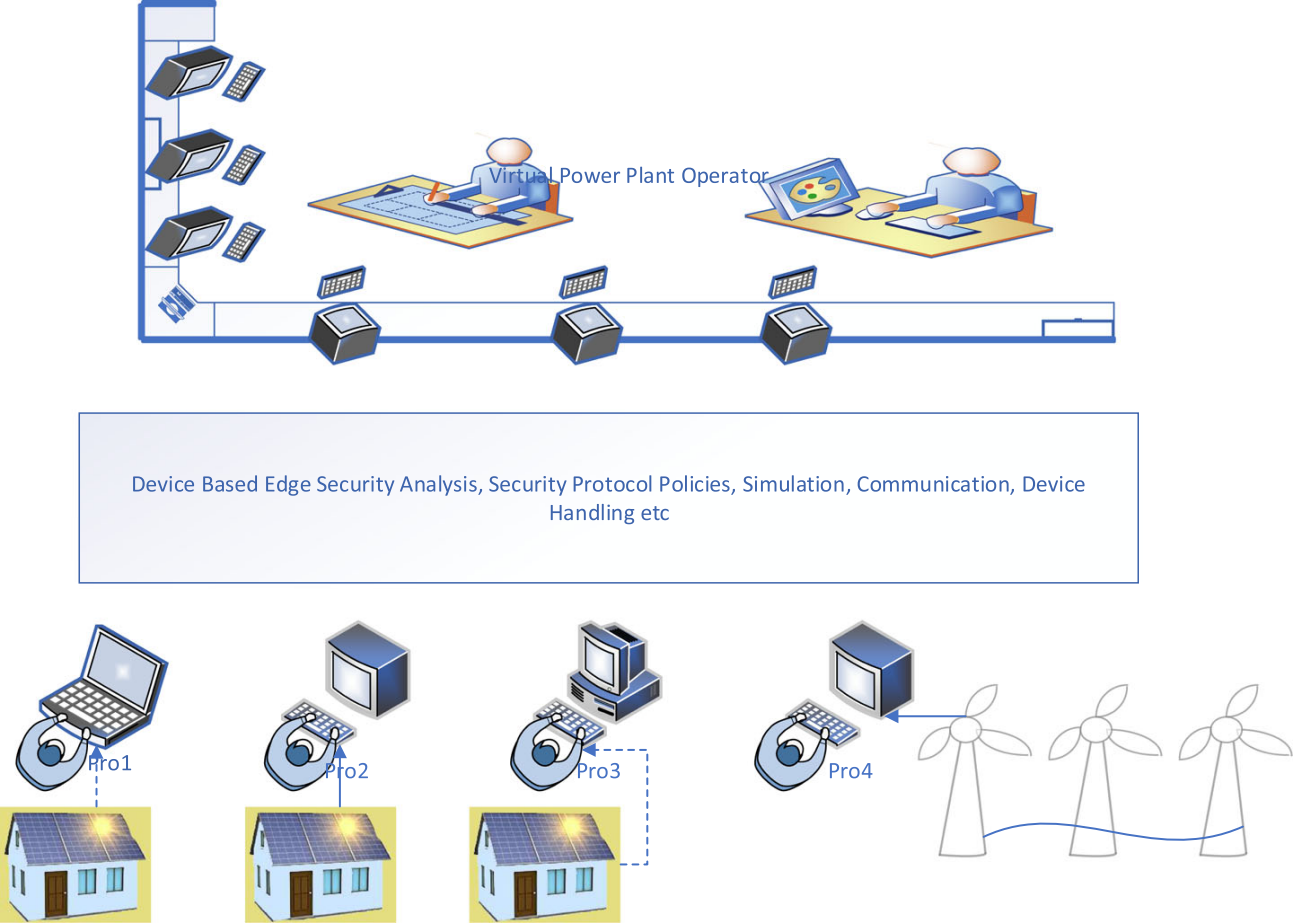
Device-Centric Edge Security for VPP

Figure 5 shows Device-Centric Edge security for Virtual Power Plant based on EdgeSec Model. In this model, each prosumer registers the devices with a specific security profile managing the module. The prosumer specific security details are then collected, and device-specific requirements are then identified. A detailed security check is implemented carrying out particular functions, one to verify the security dependency on the specific device registered and second to deploy the security function accordingly. The Edge then identifies a suitable protocol for each of the prosumer based on the resource availability and prosumer security profile. The security simulation model in the Edge simulates the instructions before deployment. This is done to protect the safety of the virtual plant prosumer’s physical system. Other functions such as encrypting communication, coordination etc., work together.

### Firewall edge security for VPP

Edge-based firewall systems is an innovative approach to protecting resources. Hu et al. base their research using software-defined networking and suggest a comprehensive framework to detect anomalies and offer effective firewall policy resolutions accurately. This SDN based firewall has three functional components, violation detection, flow tracking and authorisation. Violation detection is handled using traditional firewall packet filtering techniques. Flow tracking is based on headers using a Header Space Analysis (HAS) tool, one of the several invariant verification tools. (Kazemian et l., 2012; Kazemian et al., 2013; Khurshid et al., 2013). The authors further define Firewall Authorisation Space to allow or deny packets based on the firewall rules, thereby enabling conversion into smaller denied and allowed spaces. On the other hand, the distributed firewall architecture is placed at the network edge and adopts a master-slave architecture, thereby providing centralised management (Markham et al., 2001).

Most prosumers in a virtual power plant are small-time operators and cannot support huge firewalls or necessary infrastructure to support them. Assuming that a single virtual power plant operator has a considerable number of generators connected, it will be too costly to manage the installation of firewalls.

**Fig. 6:**
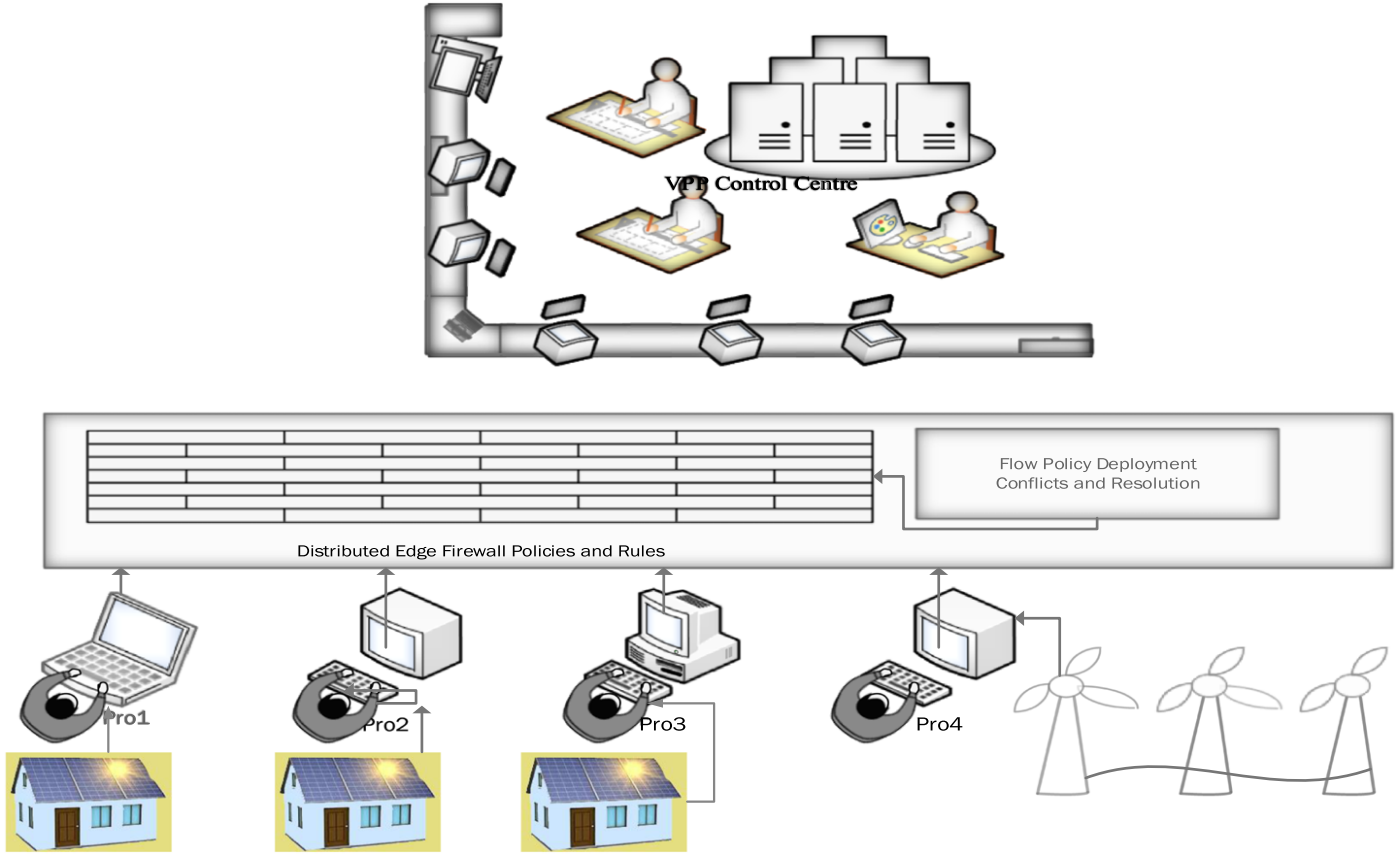
Distributed Edge Based Firewall

Figure 6 describes an edge-based firewall design. The firewall policies are converted into flow policies. The conflictions in these policies are resolved and later applied as a firewall rule. These firewall rules are applied in the edge layer. The incoming and the outgoing traffic out of the individual prosumers/consumers are examined and later allowed or disallowed. The edge-based firewalls are feasible and easier to deploy. The managing of the firewall is also easy as there is only one centralised firewall. Further, the system can be modified to suit the need-base security model.

### Edge-based intrusion detection systems (EIDS)

According to security researchers, the energy sector is the most frequently targeted sector by cybercriminals. As of 2019, 16% of the attacks were concentrated on energy with advance attacks and remained at the top 10 targeted industries (Kreyenber, 2019). The recent DDoS attacks in 2016 caused significant losses (Brewster, 2016). The availability of a distributed intrusion detection could significantly have enabled the security researchers to detect these type of security attacks at an early stage and prevent it (Sha et al., 2020). The availability of the information in this makes a vital difference. Researchers. The use of A.I. and machine learning algorithms in the security layer could significantly change the dynamics of security due to learning from multiple sources. The ease of adaptability to the changing scenarios could make a huge difference. Some notable research in apply edge-based IDS is discussed in papers by several researchers Yaseen et al. (Yaseen et al., 2016). (Roman et al., 2018). (Haddadi et al., 2018). (Roman et al., 2018) suggest a VIS (Virtual Immune System) to analyse network traffic with two functions: the kernel and the immune cells. The orchestrator inside the kernel is used for the configuration and deployment of the immune cells. The immune cells scan, analyse, manages the traffics and is also responsible for storing logs. Haddadi et al., in their research paper on SIOTOME, illustrate Edge-based architecture for IoT security. Here, the edge data collector is used for monitoring the network traffic information in the IoT devices. The edge layer analyses the traffic collected information on network threats, attacks, and feedback on the controller’s collected information. The SIOTOME also enables the defence mechanism like network isolation (Nunes et al. 2014), limiting the attack surface area. They also aid in stopping vulnerability scans and DDoS attacks.

**Fig. 7:**
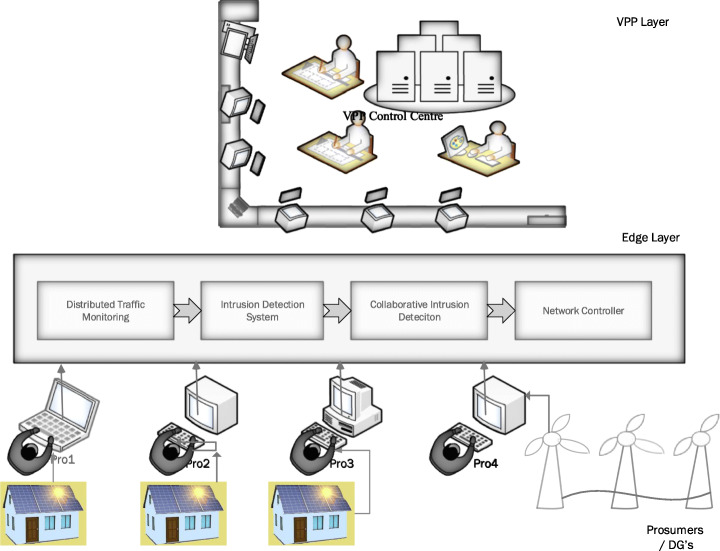
Edge Based Intrusion Detection System (EIDS)

**Fig. 8:**
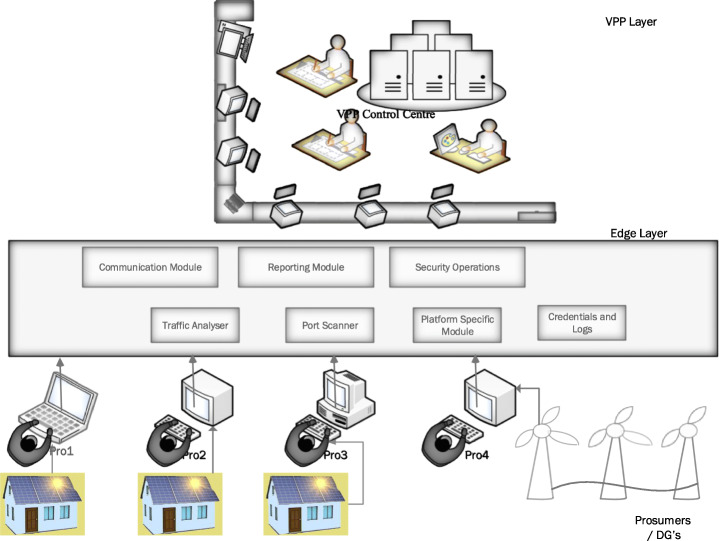
Edge-based Virtual Immune System

Figure 7 and Fig. 8 shows a simple Edge-based IDS system design and Virtual Immune System. The DTM (Distributed Traffic Monitoring System) collects the information from the individual prosumers in real-time. The system then runs the intrusion detection algorithms. There is a collaborative compilation of the traffic, and the results are then enforced on to the network controller.

### Edge-based authentication and authorisation in virtual power plants

Industrial Control system attacks in the energy sector have witnessed a surge in recent times (Wilhoit et al., 2013; Dasgupta et al., 2017). This brings into focus two main features, authentication and authorisation, which can unauthorised attacks and DDoS attacks (Kolias et al., 2017). The drawback in the devices using end to end communication is difficult to create due to heteromerous peers. Secondly, signature-based algorithms can only be employed in the traditional authentication mechanism, making it difficult to apply in virtual power plant areas. The insertion of an Edge layer improves the prospects of utilising multi-authentical protocols and multiple phase authorisation. Sha et al., in their paper, discuss the Edge-based device as a mutual authenticator with a two-phase authentication protocol. In the first stage, the edge authenticator authenticates using a digital signature and gathers users credentials. The credentials obtained are then reauthenticated using a mutual authenticator using a symmetric key-based algorithm (Sha et al., 2014; Sha et al., 2017). Researchers have also attempted to enhance the authentication protocols using RFID based algorithms. (Fan et al., 2012; Gope et al., 2018).

The process of authenticating prosumers in a virtual power plant is segmented, including the prosumers end devices and the edge layer. Depending on the characteristics of the communication, the protocols can be customised. Thus, the Edge layer works as the man in the middle, which helps set up mutual authentication and authorisation. As the Edge provides multiple authentication interfaces; thus, it provides a secure interface (Dasgupta et al., 2017).

### Edge-based privacy-preserving designs

Virtual power plants are a host of data hubs as prosumers and consumers contribute to power generation and attract vast cybercriminals. Data privacy takes precedence and requires stringent policies, monitoring and protection. As more and more devices get connected to virtual power plant operators, the data available to the plant operators is vast and needs to be protected from both the prosumer and operator levels. It is possible to achieve greater privacy by adapting different privacy protection algorithms like differential privacy (Dwork, 2014), k-anonymity (Sweeney, 2002; Sha et al., 2006; Xi et al., 2007), privacy preservation aggregation (Lu et al., 2017) etc.

Lu et al., in their paper on privacy protection, suggest a method to keep the privacy intact by using a lightweight privacy-preserving data aggregation scheme for IoT devices. They use a message authentication code to process the information reported by the devices. Once the Edge receives the authenticate of the devices by comparing the MAC and then generate a value for the IoT applications. Gentry, in his thesis, for solving a cryptographic problem, present fully homomorphic encryption. They use a simple algorithm based on a bootstrap mechanism for encryption through a recursive self-embedding algorithm “Paillier” (Gentry, C, 2009). One way hashing technique and the Chinese remainder theorem have also been used to address the privacy problem (Pei et al., 1996; McSherry & Talwar, 2007).

**Fig. 9:**
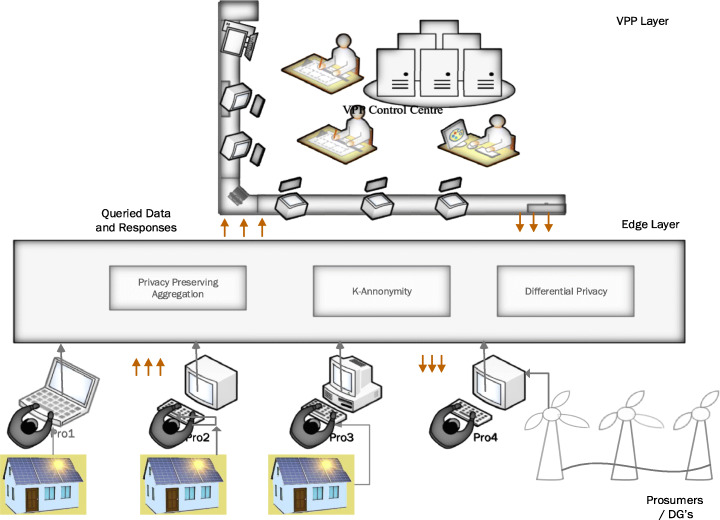
Edge-Based Privacy-Preserving Model

Figure 9 shows a brief overview of applying Edge design for preserving privacy. The Edge architecture uses privacy-preserving aggregation, k-anonymity and differential privacy together to decipher the queried data and responses between the prosumers and virtual plant operators to ensure data protection at either end. Data transmitted is verified, authenticated and established, thus ensuring privacy protection.

## Discussion

The previous section portrays different research techniques that have been applied in different platforms and suggest applications in virtual power plant areas. The Edge computing methods are still in their infancy, and there are still numerous challenging issues that need to be addressed. Though the Edge layer provides a new model for providing security solution, the Edge has a vast surface area and could, in turn, be subjected to attack. Addressing the security concerns in the Edge layer is not a huge task as opposed to other data centre securities. Thus, warranting more research in the area.

Though there are several Edge-based privacy protection techniques, the Edge protocols applied may, in turn, start to track the data and may have vested interests. (Razeghi & Voloshynovski, 2018) (Sharma & Chen, 2017). This in-turn, will warrant other innovative security solutions for protecting privacy. Studies have been carried out using Isolation techniques, but it remains to be seen how to implement the techniques in the edge layer effectively. It also remains to be seen how to effectively adopt new algorithms to establish trusted security between the Edge devices and the prosumer devices. Researchers have also proposed adopting machine learning algorithms to advance researches in intrusion detection techniques. Buczak et al. present a survey on using data mining and machine learning techniques as methods for intrusion detection. (Buczak & Guven, 2016). The popularity of deep learning has also contributed to understanding intrusion detection (Yin et al., 2017). However, the machine learning algorithmic methods require huge data sets and are most central to the environment and hence is a drawback for deployment in small Edge environments. Secondly, machine learning algorithms are more suited and beneficial in the cloud. This provides us with an opportunity to research and deploy cross-domain algorithms for intrusion detection.

Machine learning algorithms are learners, and they learn from the different attack detection techniques employed for intrusion detection. Therefore, the returned data has to be accurate and correct, on which decisions are based (Sha & Zeadally, 2015). However, there is a lack of data protocols to analyse and ensure the correctness of a high-quality dataset. In this environment, cross-domain verifications would be of great interests. (Sha et al., 2010). There has been a little contribution towards researching the cost impacts in the Edge environment. Research in the cost-benefit analysis of deploying Edge should be encouraged with active participation and collaboration. Though the safety of the prosumer equipment is extremely important, the research in this field is limited to a few. As virtual power plants are real-time, the requirements are real-time, thus complicating the simulations and modelling a suitable design (Weber & Studer, 2016). This also poses a challenge for response time to potential safety risks to minimise damages caused towards the equipment etc.

Virtual machines have found widespread use in many areas, and it is being researched in the application of the Edge layer. The ease of deploying V.M.s in the environment also pose a security threat as more than one V.M. could be deployed in the layer (Tsai, 2012; Eldefrawy et al., 2017). Considering the virtual power plant environment, these machines need to be simple, light and should meet the requirements of the prosumers. Thus, there is a huge scope for researching in this area.

## Remarks and conclusions

The challenge of securing virtual power plants systems has generated great interests among researchers. The nature and operations of the virtual plants and prosumer/consumer generators pose significant challenge and risks. The advancement of new technologies in computing like edge computing has resulted in researching edge-based security systems for virtual power plants and distributed generators. This paper aims to present an assessment and a way of adopting Edge-based security systems in virtual power plants. In this context, it has defined to provide Edge-centric architecture. These solutions aim to address key protection of VPP devices, including a comprehensive cybersecurity architecture, application of Edge-based firewalls, intrusion detection systems, Edge-based authentication and authorisations.

## Abbreviations

IoE: Internet of Energy
DDoS: Distributed Denial of Service
RTU: Remote Terminal unit
MTU: Master Terminal Unit
SCADA: Supervisory Control and Data Acquisition
TSO: Transmission System Operator
DSO: Distribution System Operator
AMI: Advanced Metering Infrastructure
A.I: Artificial Intelligence
